# Ex-PRESS Implantation versus Trabeculectomy in Open-Angle Glaucoma: A Meta-Analysis of Randomized Controlled Clinical Trials

**DOI:** 10.1371/journal.pone.0086045

**Published:** 2014-01-23

**Authors:** Guohai Chen, Wensheng Li, Fangzheng Jiang, Sihong Mao, Yuhua Tong

**Affiliations:** 1 Department of Ophthalmology, Quzhou People's Hospital, Quzhou, Zhejiang, PR China; 2 Xiamen Eye Center of Xiamen University, Xiamen, Fujian, PR China; University of Utah, United States of America

## Abstract

**Objective:**

To evaluate the efficacy and safety of Ex-PRESS implantation (Ex-PRESS) compared to trabeculectomy in the treatment of patients with open-angle glaucoma (OAG).

**Methods:**

A comprehensive literature search using the Cochrane Methodology Register to identify randomized controlled clinical trials (RCCTs) comparing Ex-PRESS to trabeculectomy in patients with OAG. Efficacy estimates were measured by weighted mean difference (WMD) for the percentage intraocular pressure reduction (IOPR%) from baseline to end-point, and odds ratios (OR) for the complete success rate and postoperative interventions. Safety estimates were measured by OR for postoperative complications. Statistical analysis was performed using the RevMan 5.1 software.

**Results:**

A total of four RCCTs were selected for this meta-analysis, including 215 eyes of 200 patients (110 eyes in the Ex-PRESS group, 105 eyes in the trabeculectomy group). There was no significant difference between Ex-PRESS and trabeculectomy in the IOPR% (WMD = 3.15; 95% confidence interval (CI), −6.17–12.47; P = 0.51). The pooled OR comparing Ex-PRESS to trabeculectomy for the complete success rate at one year after surgery were in favor of Ex-PRESS (OR = 2.93; 95% CI, 1.39–6.16; P = 0.005). The Ex-PRESS procedure was found to be associated with lower number of postoperative interventions (OR = 0.23; 95% CI, 0.07–0.81; P = 0.02) and with a significantly lower frequency of hyphema than trabeculectomy (OR = 0.21; 95% CI, 0.05–0.85; P = 0.03), whereas other complications did not differ statistically.

**Conclusion:**

In OAG, Ex-PRESS and trabeculectomy provided similar IOP control, but Ex-PRESS was more likely to achieve complete success, with fewer postoperative interventions. Complication rates were similar for the two types of surgery, except for a lower frequency of hyphema in the Ex-PRESS group.

## Introduction

Glaucoma is a leading cause of blindness. It has been estimated that over 5.9 million people worldwide will be bilaterally blind with open-angle glaucoma (OAG) by 2020, which is more prevalent in Europe and China compared to other parts of the world [1]. OAG is a progressive optic neuropathy, resulting in loss of retinal ganglion cells leading to progressive damage of the visual field. Glaucoma treatments are directed at reducing intraocular pressure (IOP), either pharmacologically or surgically. Glaucoma filtering surgery is required when IOP can no longer be controlled with medication or laser treatment.

Trabeculectomy has been the standard IOP-lowering procedure for OAG. Its success rate and complications are well established [2], [3]. The Ex-PRESS miniature glaucoma device implantation (Ex-PRESS) has been developed as an alternative filtration procedure to trabeculectomy. Initially, the Ex-PRESS device was designed to be implanted at the limbus directly under the conjunctiva. However, it was found that this mehtod leads to high rate of complications, such as hypotony and conjunctival erosion [4], [5]. The procedure was modified for the device to be placed under a partial thickness scleral flap [6]. Ex-PRESS has the potential advantage of being less traumatic than trabeculectomy as there is no need for an iridectomy and no removal of scleral tissue.

The efficacy and safety of the Ex-PRESS compared to trabeculectomy has been studied before, with the majority of reports suggesting similar efficacy and complication rates for the two types of surgery [7]–[9]. A recent meta-analysis of controlled clinical trials indicated that Ex-PRESS was recommended for uncontrolled glaucoma [10]. To our knowledge, there has been no meta-analysis of randomized controlled clinical trials (RCCTs) comparing the outcomes of Ex-PRESS versus trabeculectomy in patients with OAG. Therefore, we undertook a meta-analysis of all available RCCTs to assess the efficacy and safety of these two surgical procedures for the management of OAG.

## Materials and Methods

### Search Strategy

We conducted searches of PUBMED, EMBASE, ISI Web of Science, and the Cochrane Library, using the terms *Ex-PRESS, glaucoma* and *trabeculectomy*. A manual search was performed by checking the reference lists of original reports and review articles to identify studies not yet included in the computerized databases. The final search was carried out on August 2013, without restrictions regarding publication year or language.

### Inclusion and Exclusion Criteria

Articles were considered eligible for inclusion in the meta-analysis if the studies met the following inclusion criteria: (i) study design: RCCT; (ii) population: OAG patients (minimum age of 18 years with OAG that could not be controlled with maximal tolerated medical therapy and without history of uveitis or other ocular abnormality); (iii) intervention: Ex-PRESS versus trabeculectomy; (iv) outcome variables: at least one of the outcomes of interest discussed below was included. Abstracts from conferences and full texts without raw data available for retrieval, duplicate publications, letters, and reviews were excluded. For sequential reports on the same cohort of patients, only the most recent report was included, and data that could not be obtained from this last publication were obtained from the previous reports.

### Outcome Measures

For efficacy, the primary outcome measure was the percentage of the IOP reduction (IOPR%). When authors reported the mean value and the standard deviation (SD) of the IOPR%, we used these values directly. For studies that only reported absolute values for the IOP at baseline and at end-point, the IOP reduction (IOPR) and the SD of the IOPR (SD_IOPR_) were calculated as follows: IOPR = IOP_baseline_−IOP_end-point_, SD_IOPR_ = (SD_baseline_^2^+SD_end-point_^2^−SD_baseline_×SD_end-point_)^1/2^, then the IOPR% and the SD of the IOPR% (SD_IOPR_%) were estimated by IOPR% = IOPR/IOP_baseline_, SD_IOPR_% = SD_IOPR_/IOP_baseline_ [11]. The secondary outcome measure was the proportion of patients with complete success at one year after surgery, which was defined as target end-point IOP (<18 mmHg) without anti-glaucoma medication. The tertiary outcome was the proportion of patients with postoperative interventions (e.g., bleb needing). We assessed safety by considering the proportions of patients with postoperative complications, including hypotony, shallow or flat anterior chamber, choroidal effusion, hyphema, and encapsulated bleb.

### Data Extraction

The data were extracted independently by two reviewers (G.H.C. and W.S.L.). Disagreement was resolved by discussion. The information extracted from each study included the authors of each study, the year of publication, information on study design, location of the trial, duration of the study, number of subjects, IOP measurements, and success rate. The proportion of patients with postoperative interventions and complications was also recorded.

### Qualitative Assessment

The qualities of RCCTs were assessed by two independent observers (F.Z.J. and S.H.M.) using a system reported by Downs and Blacks [12]. The system comprises 27 items distributed between 5 subscales regarding reporting (10 items), external validity (3 items), bias (7 items), confounding (6 items), and power (1 item). Any discrepancy in the qualitative assessment between the two observers was discussed and a consensus was reached. The total score of each trial was expressed as a percentage of the maximum achievable score. The studies with a quality score>50% were considered to have adequate quality.

### Statistical Analysis

The quantitative data were entered into Cochrane Review Manager (RevMan, software version 5.1, Copenhagen, Denmark: The Nordic Cochrane Center, The Cochrane Collaboration, 2011). For continuous variables (e.g., IOPR%), the weighted mean difference (WMD) was measured, while the odds ratios (OR) were measured for dichotomous variables (e.g., number of eyes). Both outcomes were reported with a 95% confidence interval (CI). P<0.05 was considered statistically significant on the test for overall effect. The I^2^ statistic was calculated to assess heterogeneity between studies (P<0.05 was considered representative of significant statistical heterogeneity). If there was heterogeneity between studies, a random-effects model was applied to the data. Alternatively, a fixed effects model was used for pooling the data. Begg's rank correlation test and Egger's linear regression test were employed to quantitatively assess publication bias (P<0.05 was considered representative of significant statistical publication bias).

## Results

### Overall Characteristics of Selected Trials and Quality Assessment

**Figure 1:**
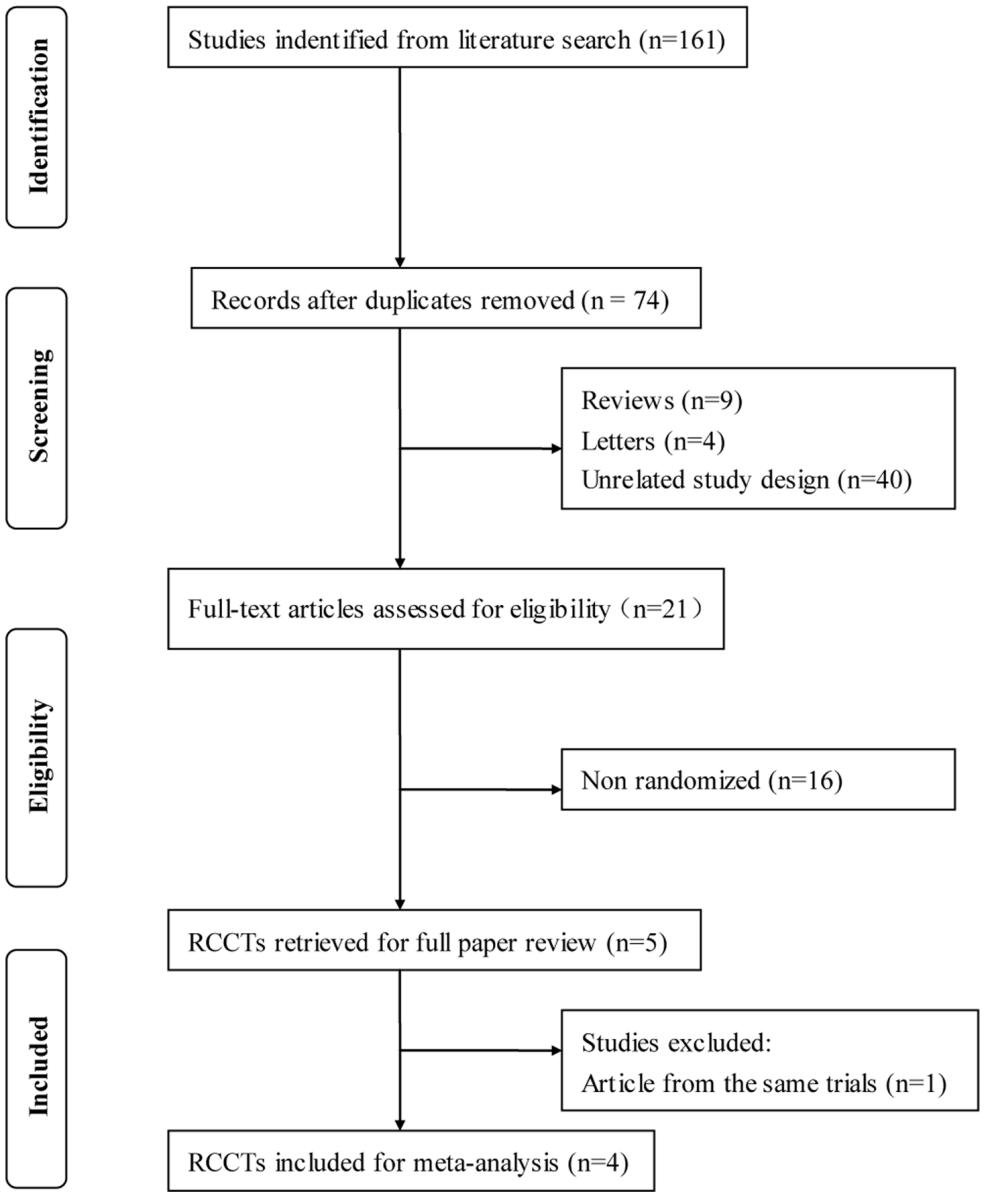
Flow diagram of studies included in this meta-analysis. RCCT = randomized controlled clinical trial.

A total of 74 articles were initially identified. Of these, 69 were rejected according to the exclusion criteria listed above. The five remaining articles with full texts that met the inclusion criteria were assessed [13]–[17]. Two articles were from the same clinical trial, and the most recent article was selected [13], [14]. Hence, a total of 4 studies were included in this meta-analysis. Figure 1 provides a flow diagram of the search results. In total, there were 215 eyes of 200 patients included in this meta-analysis. 110 eyes were included in the Ex-PPESS group, and 105 eyes were included in the trabeculectomy group. All studies fulfilled the quality criteria (Downs and Blacks score) of over 50%. The characteristics of the studies included and quality scores are summarized in Table 1.

**Table 1:** Characteristics and quality scores of included studies.

First author (year)	Design	Location	Follow-up(mo)	No. eyes*	Age (year)*	Quality score(%)
Beltran-Agullo (2013)	RCCT	Canada	6	33/31	65.9/61.9	68.75
Dahan (2012)	RCCT	South Africa	30	15/15	65.4/65.4	75.00
de Jong (2011)	RCCT	The Netherlands	60	39/39	62.4/68.6	75.00
Patel (2013)	RCCT	Canada	12	23/20	18∼85**	65.63

* Ex-PRESS implantation group/Trabeculectomy group;

** Without mean age records; RCCT = randomized controlled clinical trial.

### Efficacy Analysis

**Figure 2:**
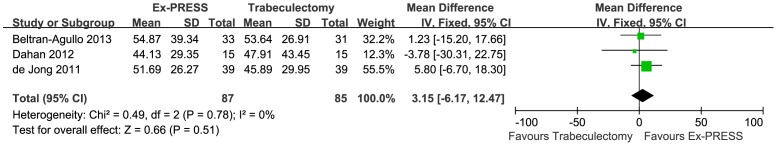
Percent intraocular pressure reduction from baseline comparing Ex-PRESS to trabeculectomy. SD = standard deviation; IV = inverse variance; CI = confidence interval; Ex-PRESS = Ex-PRESS implantation.

**Figure 3:**
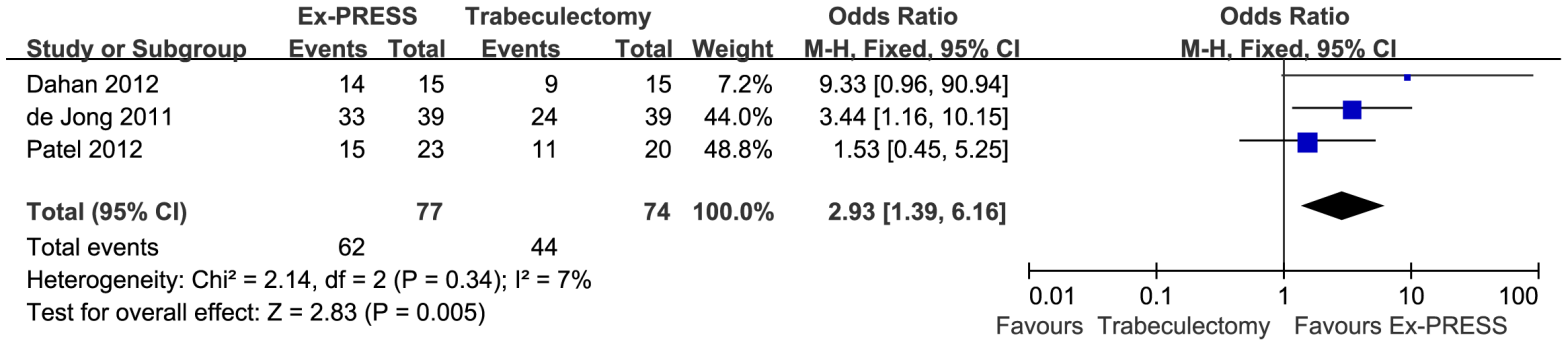
Complete success at one year after surgery comparing Ex-PRESS to trabeculectomy. M-H = Mantel-Haenszel; CI = confidence interval; Ex-PRESS = Ex-PRESS implantation.

**Figure 4:**
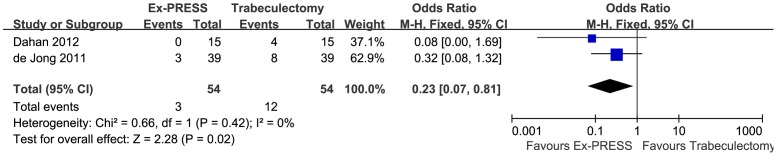
Postoperative interventions comparing Ex-PRESS to trabeculectomy. M-H = Mantel-Haenszel; CI = confidence interval; Ex-PRESS = Ex-PRESS implantation.

Three studies involving 172 eyes compared Ex-PRESS to trabeculectomy in terms of the IOPR%. The combined results showed that both surgical procedures significantly decreased the IOP. There was no significant difference between Ex-PRESS and trabeculectomy in the IOPR% (WMD = 3.15; 95%CI, −6.17–12.47; P = 0.51), with no heterogeneity identified (I^2^ = 0%; P = 0.78) (Figure 2). Complete success at one year after surgery was achieved in 62 of 77 patients in the Ex-PRESS group compared with 44 of 74 patients in the trabeculectomy group. The pooled OR comparing Ex-PRESS to trabeculectomy for the complete success rate at one year were in favor of Ex-PRESS (OR = 2.93; 95% CI, 1.39–6.16; P = 0.005), with no heterogeneity identified (I^2^ = 7%; P = 0.34) (Figure 3). Of the two studies that reported postoperative interventions, a significant difference was found between Ex-PRESS and trabeculectomy (OR = 0.23; 95% CI, 0.07–0.81; P = 0.02), with no heterogeneity identified (I^2^ = 0%; P = 0.42) (Figure 4). Begg's rank correlation test and Egger's linear regression test indicated no publication bias for any of the parameters.

### Safety Analysis

**Figure 5:**
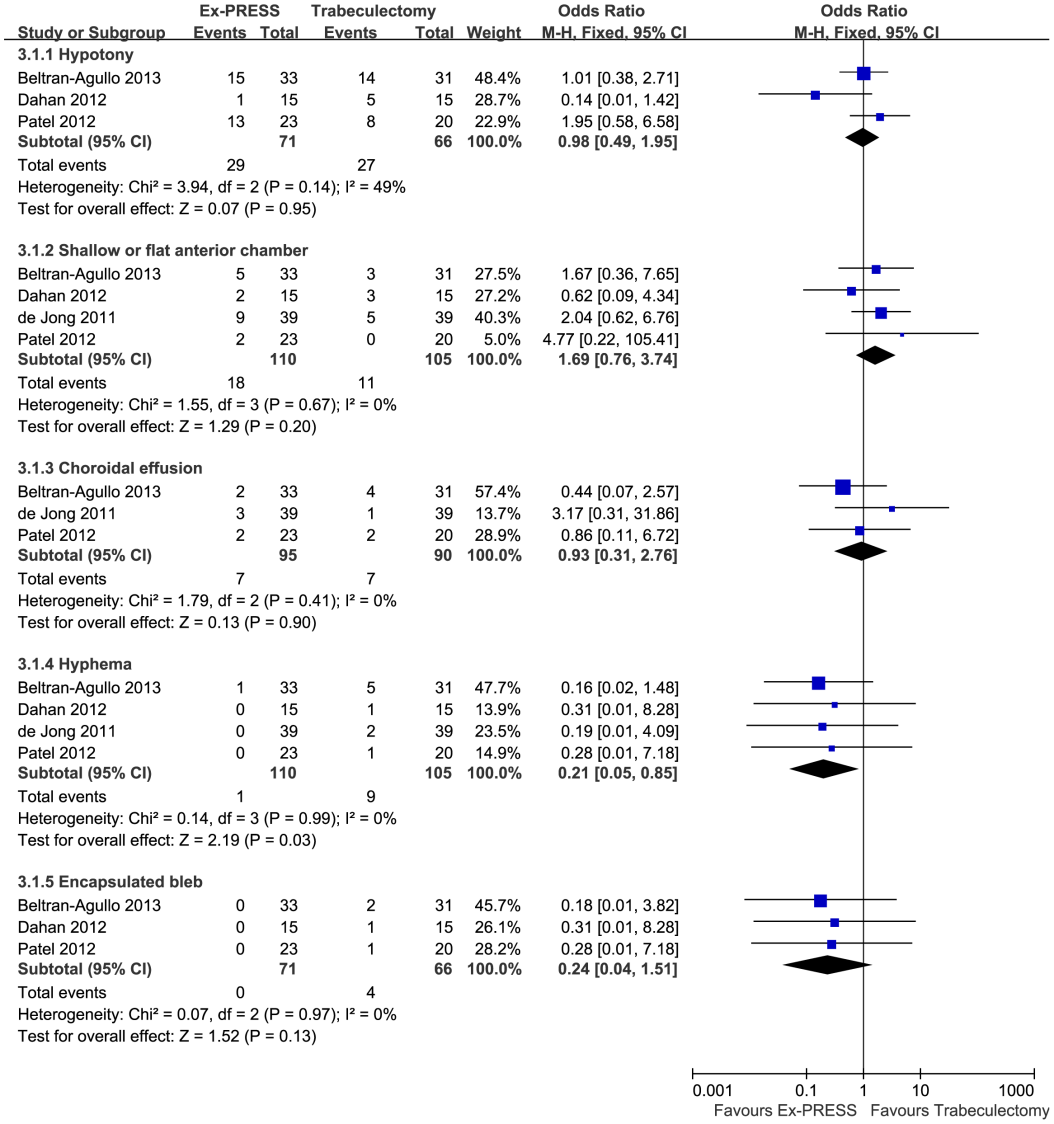
Postoperative complications comparing Ex-PRESS to trabeculectomy. M-H = Mantel-Haenszel; CI = confidence interval; Ex-PRESS = Ex-PRESS implantation.

Postoperative complications comparing Ex-PRESS to trabeculectomy are shown in Figure 5. The three most common postoperative complications were hypotony, shallow or flat anterior chamber, and choroidal effusion. There were no significant differences between Ex-PRESS and trabeculectomy with respect to the incidence of hypotony, shallow or flat anterior chamber, or choroidal effusion (OR = 0.98; 95%CI, 0.49–1.95; P = 0.95; OR = 1.69; 95%CI, 0.76–3.74; P = 0.20 and OR = 0.93; 95%CI, 0.31–2.76; P = 0.90, respectively), and no statistical heterogeneity was observed between the studies (P = 0.14, P = 0.67, and P = 0.41, respectively). The Ex-PRESS was associated with a significantly lower frequency of hyphema than trabeculectomy (OR = 0.21; 95% CI, 0.05–0.85; P = 0.03), with no heterogeneity identified (P = 0.99). The Ex-PRESS was associated with a lower frequency of encapsulated bleb, but a difference that was not statistically significant (OR = 0.24; 95%CI, 0.04–1.51; P = 0.13), with no statistical heterogeneity (P = 0.97).

## Discussion

Glaucoma treatments are directed at reducing IOP. Traditionally, trabeculectomy is considered the standard IOP-lowering procedure for OAG. Ideally, any procedure seeking to replace trabeculectomy should offer at least the same success rate, without increasing the level of complications. In recent years, Ex-PRESS has been introduced as an alternative to trabeculectomy. Numerous studies have reported on the biocompatibility, safety, and efficacy of Ex-PRESS during its evolution over the last decade [18]–[20]. However, trials have usually shown conflicting results, making it difficult to draw conclusions.

In this meta-analysis, we reviewed four RCCTs, including 215 eyes of 200 patients (110 eyes in the Ex-PRESS group, 105 eyes in the trabeculectomy group). In assessing the IOP, Ex-PRESS was associated with IOP-lowering efficacy comparable to that of trabeculectomy, demonstrating slightly bigger IOPR% reduction from baseline, but this difference that was not statistically significant(WMD = 3.15; 95%CI, −6.17–12.47; P = 0.51). Our analysis of the IOPR% was based on data pooled from trials of different durations, ranging from six months to five years. The longest follow up was five years by de Jong et al [14], which reported Ex-PRESS with better IOP control during the first three years, matching the trabeculectomy IOR lowering effect at four and five years. It was a compromise proposal to choose the data from the end-point, owing to the lack of data reported in all phases of follow-up and trials with different durations. With respect to the complete success rate at one year, Ex-PRESS was more likely to achieve complete success (OR = 2.93; 95% CI, 1.39–6.16; P = 0.005), with fewer postoperative interventions (OR = 0.23; 95% CI, 0.07–0.81; P = 0.02). The success rate during the first three years after Ex-PRESS was significantly higher, differences did not reach statistical significance up to year 3 reported by de Jong et al [14], which was the longest follow up. As only one year follow up results were available in three of the four trials included, our meta-analysis was focused on success rate at one year post surgery. For safety, Ex-PRESS was associated with a significantly lower frequency of hyphema than trabeculectomy (OR = 0.21; 95% CI, 0.05–0.85; P = 0.03), whereas other complications did not differ statistically. The Ex-PRESS procedure did not require an iridectomy, which is commonly performed with trabeculectomy, possibly resulting in an increased likelihood of inflammation and hyphema [21].

This meta-analysis may have some limitations. First, we cannot fully exclude publication bias. Although the Begg's and Egger's tests demonstrated no evidence of publication bias, the results should be interpreted with caution. Second, the studies were carried out with small or very small sample sizes and it is impossible to mask the surgical technique as trabeculectomy is easily differentiated from Ex-PRESS during postoperative follow-ups. These factors can affect the interpretation of the results. Third, a potential source of heterogeneity is trials duration and lack of data reported in all phases of follow-up.

Our findings indicate that Ex-PRESS and trabeculectomy provide similar IOP control, but Ex-PRESS is more likely to achieve complete success, with fewer postoperative interventions. Complication rates were similar for the two types of surgery, except for a lower frequency of hyphema noted in the Ex-PRESS group. RCCTs with longer duration and larger sample size are needed to provide more definitive information indicating whether Ex-PRESS is an effective and safe procedure for the treatment of OAG.

## Supporting Information

Checklist S1 **PRISMA Checklist.**
(DOC)
